# Forensic genetics through the lens of Lewontin: population structure, ancestry and race

**DOI:** 10.1098/rstb.2020.0422

**Published:** 2022-06-06

**Authors:** Mark A. Jobling

**Affiliations:** Department of Genetics and Genome Biology, University of Leicester, University Road, Leicester LE1 7RH, UK

**Keywords:** Richard Lewontin, apportionment of genetic diversity, human genetics, forensic genetics, biogeographic ancestry prediction, externally visible characteristics

## Abstract

In his famous 1972 paper, Richard Lewontin used ‘classical’ protein-based markers to show that greater than 85% of human genetic diversity was contained within, rather than between, populations. At that time, these same markers also formed the basis of forensic technology aiming to identify individuals. This review describes the evolution of forensic genetic methods into DNA profiling, and how the field has accounted for the apportionment of genetic diversity in considering the weight of forensic evidence. When investigative databases fail to provide a match to a crime-scene profile, specific markers can be used to seek intelligence about a suspect: these include inferences on population of origin (biogeographic ancestry) and externally visible characteristics, chiefly pigmentation of skin, hair and eyes. In this endeavour, ancestry and phenotypic variation are closely entangled. The markers used show patterns of inter- and intrapopulation diversity that are very atypical compared to the genome as a whole, and reinforce an apparent link between ancestry and racial divergence that is not systematically present otherwise. Despite the legacy of Lewontin's result, therefore, in a major area in which genetics coincides with issues of public interest, methods tend to exaggerate human differences and could thereby contribute to the reification of biological race.

This article is part of the theme issue ‘Celebrating 50 years since Lewontin's apportionment of human diversity’.

## 1. Introduction

**Figure 1.**
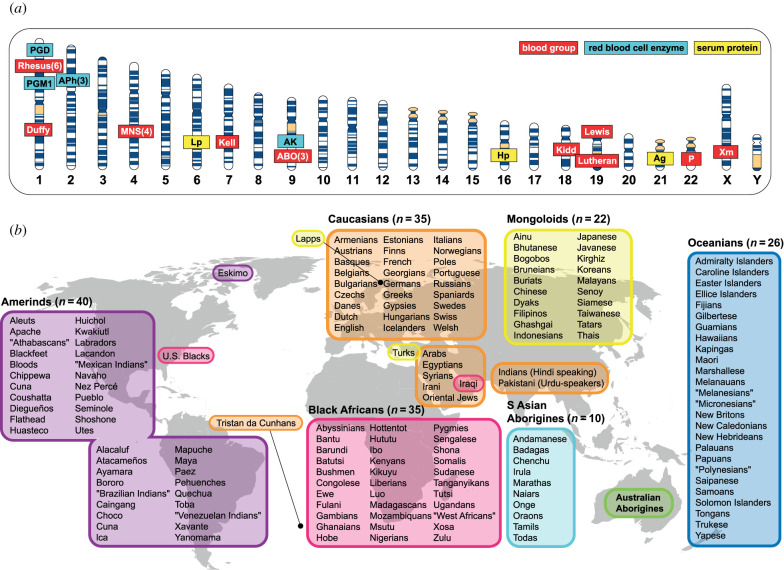
Markers and populations used in Lewontin's ‘The apportionment of human diversity’. (*a*) Lewontin's [1] 17 ‘classical’ markers are shown in their approximate chromosomal locations (from www.omim.org) on a G-banded human karyotype. Thirteen of the markers were diallelic; for the remaining four, the number of alleles analysed is given in parentheses after the marker name. All markers are also among those used in forensic serological analysis [2]. APh: acid phosphatase 1; AK: adenylate kinase 1; PGM1: phosphoglucomutase 1; PGD: phosphogluconate dehydrogenase; Ag: β-lipoprotein, Ag system; Lp: β-lipoprotein, Lp system; Hp: haptoglobin. (*b*) Lewontin's [1] 169 populations are shown, with assignment to one of seven racial groups indicated by background colour (*n* indicates number of populations per racial group). Not all populations were typed for all 17 markers shown in (*a*). Sets of populations are placed on the world map to indicate approximate regions of origin; north and south Native Americans are distinguished here, though were considered as one ‘Amerind’ group by Lewontin. For some populations, geographical location and racial group assignment indicate anthropological classifications and some examples (e.g. US Blacks, Turks) are placed separately from the major sets. Names of populations and racial groups are those given by Lewontin [1]; the significance of inverted commas round some population names is unclear.

When Richard Lewontin wrote his seminal 1972 article, ‘The apportionment of human diversity’ [1], he had at his disposal extensive molecular population data based on an array of 17 ‘classical’ polymorphisms (figure 1*a*), detectable by protein electrophoresis or immunological methods, that allowed him to assess variation within and between human groups. Lewontin found that 85.4% of total human diversity was contained within populations, and he emphasizes his point that ‘less than 15% of all human genetic diversity is accounted for by differences between human groups’ with an exclamation mark. However, in 1972, these same polymorphic markers formed the basis of another field, with a different aim: attributing a biological sample to an individual [2]. That field is now known as forensic genetics. Taking Lewontin as a starting point, this review examines how human individual identification evolved from ‘classical’ polymorphisms to DNA, how it attempted to account for inter-population variation and population structure, and how, in no-suspect cases where database searches draw a blank, it has considered the apportionment of human diversity to make deductions about the population of origin of a sample (‘biogeographic ancestry’; BGA).

The lens of Lewontin allows us to see how unchanging and intractable some of the problems are: what characteristics we use to classify populations, how we name them and how they should be grouped in higher level comparisons. Lewontin's reliance on proteins, rather than DNA, brings phenotypes into play, and this leads to the uncomfortable intersection between genetic diversity and natural selection, and the characteristics, such as pigmentation, that are among the traditional racial classifiers. The fact that forensic genetics exists to serve criminal justice systems, and that such systems fail to treat all ethnic groups equitably, lends particular importance to this link with race.

## 2. The evolution of forensic genetics: from blood groups to DNA profiles

The very first known human genetic polymorphism, variation in the ABO blood group system, was identified by Karl Landsteiner in 1900 [3] and formed part of Lewontin's marker set (figure 1*a*), providing him with an ‘*embarras de richesse*’ of data [1]. From the outset, its potential in analysing blood samples from crime scenes was recognized [4]. A system such as this has low variability (few alleles and high average allele frequencies), so its power to attribute a biological sample to a particular person is correspondingly low—samples from different individuals will often have matching blood types. By contrast, a non-match is strong evidence that can exclude a suspect at just this single locus, as recognized by Landsteiner [4]: ‘to detect the non-identity of blood samples’.

**Figure 2.**
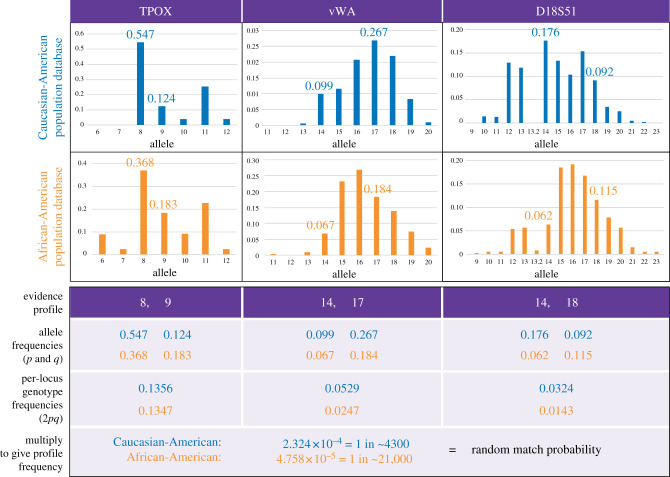
Calculation of RMPs and the effect of different population databases. Bar charts show the allele frequencies for three forensic STRs in two population databases [5], ‘Caucasian-Americans’ (*n* = 404 alleles typed) and African-Americans (*n* = 418). Note that ‘Caucasian’ is the term used by the authors but is no longer favoured in many areas of human genetics [6]. Below is an evidence profile, heterozygous at each locus, and the corresponding allele frequencies, denoted *p* and *q*. An individual can receive either allele from either parent, so the genotype probability is 2*pq* (for homozygotes, the corresponding probability is *p*^2^). Assuming the loci are independently inherited, the per-locus genotype frequencies can be multiplied together (the product rule) to give the profile frequency, which is equivalent to the RMP (the chance that some random unrelated person in the population carries the same profile as the evidential sample). In practice, many more than three STRs are analysed, giving much lower values than in this example. Given the different allele frequencies in the two databases, in this case, the profile frequency when using the Caucasian-American database is about five times higher than that for the African-American database. Note that the calculation here assumes the simplest of population genetic models (Hardy–Weinberg equilibrium) and typically in casework somewhat more complex models are used (see main text). (Online version in colour.)

Forensic biologists went on to combine sets of these classical polymorphisms to reduce the random match probability (RMP; figure 2), the chance that two different individuals have matching genotypes [2], and exploited their Mendelian inherited nature in kinship testing [7]. If genetic loci are unlinked and the population is randomly mating, then independent inheritance means that, in principle, their allele frequencies can be multiplied in deriving genotype frequencies—this is known as the product rule. As a consequence, RMPs fell to more useful average levels of 1% or lower, but there remained practical problems of protein degradation, body fluid specificity and the interpretation of mixed samples [8].

It was the development of DNA-based analysis in the mid-1980s that relegated Lewontin's classical polymorphisms from forensics and began the modern era of robust individual identification. Initially, this was via DNA fingerprinting [9], based on length variation at multi-allelic autosomal minisatellites, and by the 1990s DNA profiling [10], based on length variation at short tandem repeats (STRs; also known as microsatellites). Today, a combination of approximately 20 independently inherited autosomal STRs can provide a profile via multiplex polymerase chain reaction (PCR) from trace material, yielding RMPs as low as 10^−26^ [11].

STR profiles are digital—each allele is designated by a number reflecting its number of repeat units—and therefore ideal for databasing. This allowed the development of large investigative databases containing profiles obtained from convicted individuals, suspects and crime-scene samples. The first national DNA database to be developed (in 1995) was that of the UK [12], which by March 2020 contained 6.6 million profiles [13], the largest by proportion of population of any in the world. It provides a ‘hit’ (a match between a crime-scene profile and a stored subject profile) in 66% of queries and thus represents an efficient tool for the detection of crime.

## 3. Forensic significance of intragroup and intergroup variation

The forensic geneticist needs genotypes that provide robust individual identification, an aim that emphasizes variation within the population: calculating a RMP to evaluate the significance of a match can then be done by compiling allele frequency data from that population, and assuming homogeneity and random mating. However, this raises two issues: which population is relevant to a particular case? And can population substructure invalidate the assumptions made in RMP calculations? These questions formed some of the battle-lines in the so-called ‘DNA fingerprinting wars’ of the 1990s, in which Lewontin, together with Daniel Hartl [14], was a vigorous combatant. The debate was eventually declared settled [15], although not at all to Lewontin's satisfaction [16].

Accustomed to the compendious collections of population data on classical markers [17], Lewontin argued that a lack of similarly detailed data on the new-fangled DNA markers made RMP calculations unsound [14]. He also pointed out that the major racial groups used in calculations (for example, ‘Caucasian’) likely harboured endogamous subgroups with significantly divergent allele frequencies (sub-populations) that violated the random mating assumption and made the use of the product rule inappropriate. It was unclear whether the product rule favoured the defence or the prosecution, but in any case, it should not be applied until more detailed data were available; instead, Lewontin suggested, the profile frequency in the population database should be used, and when the profile was unobserved (i.e. in the majority of cases), a frequency of 1/*x* should be assumed, where *x* is the population database size [14]. However, clearly, without multiplication of allele frequencies at different loci, such assumed genotype frequencies were problematically and implausibly high, and undermined the utility of forensic DNA analysis [18]. Lewontin therefore lost this battle.

When a crime-scene profile matches a suspect, but the only information available about the perpetrator is the DNA profile itself, then the choice of reference population for calculating the RMP becomes particularly important. If there were major differences in allele frequencies between different populations, then a DNA profile might be very rare in one population, strongly incriminating the suspect, but orders of magnitude more common in another. The United States National Research Council (NRC) 1996 report [19] made a number of recommendations to deal with this issue that remain widely adhered to today. One is the treatment of rare alleles: when an allele is not represented in a database, or present only a few times, any estimate of its frequency is inherently inaccurate. The recommendation is that each allele should be observed at least five times if its frequency estimate is to be used in statistical calculations, and that the frequency of any allele observed less often than this should be inflated to this minimum, i.e. 5/2*N*, where *N* is the number of individuals in the database (and 2*N* the number of genomes). A second NRC recommendation was for an adjustment to account for population structure, using a correction factor known as *θ*. For US populations, a conservative value of *θ* = 0.01 is recommended—this is at least an order of magnitude higher than empirically measured values; for ‘some small, isolated populations’, a higher value of 0.03 can be used. When factored into calculations [20], *θ* has the effect of somewhat elevating genotype frequencies.

These kinds of compromises lack rigour but were justified as part of a conservative approach to statistics that favoured the defendant in a case [19]. However, declaring the end of the ‘DNA fingerprinting wars’ without solving the underlying issues means that questions of inter-population variation and population substructure have not disappeared and tend to arise afresh with each new development in forensic technology. In today's age of highly sensitive PCR multiplexes, STR profiles are often partial (missing a full set of loci, or alleles) or mixed, which can increase RMPs and make interpretation more challenging. More rigorous approaches to calculating RMPs under different models of mate-choice have been developed [21–23]. As well as considering the significance of a match between the profile of a known suspect and a crime-scene sample, in the modern world of very large investigative databases (such as those of the US and the UK) ‘cold hits’ are often reported and evaluated. In these cases, a crime-scene profile matches a profile in the database, sometimes from a case occurring long ago, when other evidence may be scanty. Here, the persuasive power of a low RMP may carry great weight, so careful calculation of the chance of erroneous matches becomes important [24].

## 4. No-suspect cases: DNA-based intelligence on ancestry

No nation holds a ‘universal’ DNA database containing the DNA profiles of all its citizens (although some have considered building one [25]). A consequence of this is that many crime-scene profiles entered into investigative databases return no hits, and therefore no potential suspects. This has led to attempts to produce intelligence from DNA information that could facilitate suspect identification. One indirect approach is the familial search [26,27]—seeking autosomal STR profiles in an investigative database that are sufficiently similar to the crime-scene profile to suggest that they could come from a close relative (parent, child or sibling). The reliability of this endeavour, like that of profile matching and database searching, is influenced by population structure [28]. The reach of the method has recently been extended to more distant relatives by generating genomewide single-nucleotide polymorphism (SNP) genotypes and using these to query publicly accessible data generated by direct-to-consumer testing [29] (investigative genetic genealogy).

Intelligence can also be sought more directly by attempting to infer characteristics of the suspect from the crime-scene sample. Here, three areas have been focused upon: BGA, externally visible phenotypes and age [30]. This review focuses on the first of these and ignores the last, since age is unaffected by variation in DNA and is more reliably assessed by measuring epigenetic variation. Because the investigated visible phenotypes show high inter-population variation and correlate with ancestry (and indeed with traditional ideas of race in contexts such as the US [31]), they intersect with the apportionment of human genetic diversity and are also considered here.

If a population geneticist today wished to study the ancestry of an unknown individual sample, they would resort to genomewide analysis via a chip typing hundreds of thousands of SNPs, or even whole-genome sequencing. But forensic scientists do not usually have this luxury, since the amount and quality of DNA available is often low [32,33]. Furthermore, the need for methods to be forensically validated, acceptable in the courtroom and compatible with existing investigative databases limits the application of genomewide techniques, and the number and type of markers that can be studied. Since standard forensic autosomal STR profiles are generated routinely, many studies have asked whether these contain any information about population of origin. More targeted work has sought SNPs with alleles that are highly differentiated between populations and therefore can have predictive value in combination.

The autosomal STRs used in DNA profiling are multi-allelic, with high mutation rates and high heterozygosity, properties that suit them to individual identification. This might be expected to make forensic STR profiles poorly differentiated between populations [8], and indeed the 13 CODIS (Combined DNA Index System) loci have a global *F*_ST_ of approximately 4.5%, measured in the highly diverse Human Genome Diversity Project (HGDP) panel of indigenous populations [34], about one third of the approximately 15% observed by Lewontin [1]. An *F*_ST_-based analysis of a large worldwide dataset based on the 13 CODIS loci indicated that these STRs systematically underestimate inter-population genetic variation [35]. However, application of the model-based clustering algorithm STRUCTURE showed that the CODIS loci give patterns of population clustering like those of other similar but independent sets of STRs [34]. This study concluded that although forensic STRs do show relatively low *F*_ST_ (a measure that is depressed for markers that are highly heterozygous [34,36]), their high heterozygosity actually strengthens ancestry inference compared to less heterozygous STR sets.

It is worth noting that the correction factor for population structure, *θ* (discussed in the section above), is equivalent to *F*_ST_ if random mating is assumed within sub-populations. The *θ* values recommended by the NRC (1% in general and 3% for ‘some small, isolated populations’) are considerably smaller than the global CODIS data (4.5% [34]); such low values are supported by analysis of forensic reference datasets [35,37], which could reflect relatively high degrees of inter-population admixture in the underlying samples.

Forensic geneticists have investigated the ancestry information contained in autosomal STR profiles and developed methods to apply this in practice. For example, a machine-learning-based tool, PopAffiliator [38], claims approximately 86% accuracy in classifying 17-locus profiles to major regions essentially representing Europe, East Asia and sub-Saharan Africa. As with many predictive methods, the output of PopAffiliator provides probabilities of membership of either three or five different large population groups, and it is left to the user as to how to interpret or report these. Among adjacent regions, classification is less reliable, as illustrated by a STRUCTURE-based clustering analysis of the HGDP panel using 15 or 20 STRs [39]: while European, African and Native American populations were highly differentiated, the HGDP populations of Europe, the Middle East and South Asia were not, and assigning a profile to one of these regions is inherently unreliable.

To build panels of markers to predict the population of origin more robustly, loci were sought that maximized allele frequency differences between populations (ancestry informative markers; AIMs). As Lewontin observed [1], such markers are atypical: the most highly differentiated example in his set of classical markers was the Duffy blood group, showing a mean of just 63.6% of its total diversity within populations, compared to an average across loci of 85.4%. The Duffy negative allele was at greater than 90% frequency in sub-Saharan African populations, but at low frequency in most others [1]. Such large differences are now taken as a signature of likely natural selection; in the case of Duffy, it was not until 3 years after Lewontin's paper that it was shown that erythrocytes from Duffy negative (now designated *FYB^ES^*/*FYB^ES^* homozygous) individuals were resistant to infection by the malaria parasite *Plasmodium vivax* [40]. This same strongly selected marker was also the most highly differentiated locus in early AIM searches in the DNA era [41], and today it persists (as the SNP rs2814778) into many current BGA SNP multiplexes in forensic use [42].

Binary AIMs were defined as variants exceeding some threshold, *δ* (the frequency of an allele in one population minus that in another; e.g. 50% [41]) in pairwise comparisons. Early on, both STRs and SNPs were included in AIM panels ([41]; with an adjustment of *δ* calculation for multi-allelic loci) but today most sets are SNP-specific. One example designed for forensic use at a global level is a panel of 55 AIM SNPs [43] that is available as a sensitive PCR multiplex. These SNPs were chosen based on their high allele frequency differences in various pairwise comparisons among a diverse collection of 63 populations. In an analysis of 3884 individuals from 73 populations using STRUCTURE, the most likely number of clusters (*K*) was eight, and the pattern of regional variation essentially resembled that observed for larger numbers of genomewide markers [44,45]. Other AIM sets have been developed for discrimination at more local levels, for example Australia and the Pacific [46], and East Asia [47]. At the individual level within populations, there can be considerable variation in cluster membership proportions so, in a likelihood-based approach, individuals can often be misassigned. If a tested individual belongs to a population that is not included in the reference set, they tend to be assigned to some geographically allied population—assignment is only as good as the reference data. Notably, the development and testing of such SNP panels is mostly based on indigenous populations that are not believed to be admixed. These may be very different from those seen in real forensic scenarios where, in urban settings, complex admixture is commonplace.

## 5. No-suspect cases: DNA-based intelligence on phenotype prediction

Traits that are forensically useful are those that a witness might observe and are collectively known as externally visible characteristics (EVCs). EVCs that are predictable from DNA variants need be largely genetically determined, and to have relatively simple genetic architecture. One such phenotype that has long been incorporated into standard DNA profiling is sex, predicted via a test for the presence or absence of the male-determining Y chromosome [48]. Beyond this, research into the genetic basis of facial shape, hair type and stature has generated long lists of variants that contribute to these complex traits, but their predictive value is too low to make them of practical forensic use [30], despite commercial offerings that promise ‘photofits’ of individuals following DNA analysis [49]. The phenotype that has received most attention is pigmentation, since this is relatively well characterized at the genetic level and variants are known that have large effects.

The global apportionment of diversity in skin colour differs from that of hair and eye colour, reflecting differing evolutionary histories. Skin colour in indigenous populations shows a globally non-random geographical distribution, with people having the darkest skin in the tropics, and those with lighter skin in more northerly regions. The most widely held theory to explain the pattern of depigmentation from the human ancestral state of dark skin is the need to synthesize vitamin D in regions of low UV radiation [50]. Following a similar methodology to Lewontin [1], Relethford quantified the apportionment of skin colour diversity [51], finding that just 9% of variation exists within populations—a reversal of the pattern found for classical markers [1], and underscoring the fact that skin colour has not evolved neutrally. By contrast, most of the global variation in hair and eye colour is among Europeans, with non-Europeans tending to show low variation; this has been taken to reflect a lack of a role for natural selection, and sexual selection has been proposed, though not proven, to be involved [50]. The different histories and patterns of these pigmentation traits have influenced the search for underlying genetic variants: since variation in hair and eye colour is maximal within a relatively homogeneous European metapopulation, association studies have been productive [52]; however, association studies for skin colour cannot easily be done across populations with different phenotypes, since the signal of ancestry obscures the phenotypic signals.

Many years of research into the genetic basis of human pigmentation [52] have yielded a collection of genes whose products govern the abundance, properties and distribution of melanin pigments, giving rise to natural variation in the colour of skin, hair and eyes. A set of 41 SNPs in a total of 19 genes (the HIrisPlex-S system [53]) now allows estimation of individual probabilities for five skin, four hair and three eye colour categories from genotypes. The predictive models were developed and validated in a set of individuals (80% of them European) from indigenous populations [53–55]; in admixed populations, while SNP-based eye and hair colour prediction perform well [56,57], skin colour prediction is less accurate [57,58], reflecting the more complex nature of this trait, and possible epistatic interactions between alleles [56].

## 6. The entanglement of population classification, ancestry and phenotype

In order to consider the apportionment of diversity among groups, we first need to define the groups. Lewontin's list of 169 populations [1] today has a retro feel to it (figure 1*b*), involved some rather arbitrary choices [59], and raises questions about how we label and classify our fellow humans. Some terms are now regarded as derogatory or politically incorrect: there are Lapps (today, Saami), Eskimos (Inuit), Gypsies (Roma) and Hottentots (probably equivalent to Khoisan). Among the Amerinds are the Blackfoot, the Bloods, the Flathead and the Nez Percé. There are labels of language (speakers of Hindi and Urdu), religion (Oriental Jews) and skin colour (US Blacks). Lewontin's seven racial classifiers (figure 1*b*) include Caucasian and Mongoloid, two of Blumenbach's eighteenth-century races.

As well as using an SNP chip, today's population geneticist would also be likely to use a classification scheme informed by ethnolinguistic affiliation, geography and subject self-definition: the 1000 Genomes Project [60] provides examples. However, in forensic practice, by contrast, analysis is carried out within the socio-political frameworks of national criminal justice systems that are rooted in their own different census populations, and often reach back into the past. Thus, the battleground of the US-focused ‘DNA fingerprinting wars’ took place among the unhelpful confusion of Caucasian, Black and Hispanic categories [61]. The first two are sometimes recast as European- and African-American; the last (derided by Lewontin as ‘a biological hodgepodge’ [14]) includes a diverse collection of Mexican, Puerto Rican, Guatemalan, Cuban, Spanish and other peoples with differing proportions of European, Native American and African ancestry. In the UK, six ‘ethnic appearance’ categories have been used [37,62]: pale-skinned Caucasian, dark-skinned Caucasian, African/African-Caribbean, Indian subcontinent, East Asian and North African/Middle Eastern. Where the lines are to be drawn between these is far from clear, and they must contain endogamous sub-populations and varying degrees of admixture. In Malaysia, there are separate population reference databases for Malay, Chinese, Indian and Orang Asli indigenous people [63]. Racial categories are context-dependent [31], rather than universal.

Pigmentation phenotypes are a hallmark of many traditional race-based classifications [64], and in forensic genetics, the conflation of pigmentation and ancestry persists not only through the way population groups are labelled but also in the markers used in ancestry testing. Two SNPs in the 55-SNP AIM set described above [43] are also part of the HIrisPlex-S prediction set [53], and two more are pigmentation associated. Other SNPs are associated with less obviously visible phenotypes: Duffy has already been mentioned, and other examples include a variant in the *EDAR* gene associated with thicker hair in Asians and a variant in the acetaldehyde dehydrogenase gene responsible for Asian alcohol flush reaction [43]. A move away from EVCs in ancestry SNP panels might help, but in practice ancestry and phenotypes are inexorably linked because the information that a DNA sample came from a European, an East Asian or an African raises expectations about the appearance and social identity of that person [65].

As well as robust prediction, an EVC has utility if it is generally rare in a population [66], since it can substantially narrow a pool of suspects. In the UK, a red hair test [67] has been available for many years and is useful because the population frequency of the trait is just 5% or so [68]. Predicted phenotypes that characterize minority ethnic populations can therefore be seen as valuable in a similar way, but are problematic in that they focus attention on groups that are often already the target of excessive police attention [69–71]. In providing a probability of belonging to a particular group or having a particular appearance, these kinds of tests point not to an individual suspect, but a pool or collective of similar suspects [72], and thus to the potential victimization of a community.

## 7. Conclusion

Lewontin [1, p. 397] notes in his 1972 paper that ‘our perception of relatively large differences between human races and subgroups, as compared to the variation within these groups, is … a biased perception and … based on randomly chosen genetic differences, human races and populations are remarkably similar to each other’. By focusing on variants that are far from random and that exaggerate the differences between populations, and by conflating ancestry and phenotypes, forensic BGA testing and the prediction of EVCs have the effect of reinforcing a link between ancestry and racial divergence that is not systematically present in the genome otherwise. Thus, despite the profound legacy of Lewontin's 1972 study [59], in a major area in which genetics coincides with issues of public engagement and interest, methods in the field tend to emphasize human differences beyond the picture that generally emerges from genetic and genomic evidence.

It would be naïve to imagine that forensic scientists will give up their efforts to maximize intelligence from DNA evidence. However, it is also important to remember that these creative endeavours are undertaken because of the absence of universal forensic DNA databases. Indeed, the biases, ethical problems and invasions of privacy that the armoury of investigative methods present have been used to bolster the arguments for universal databasing. It has been argued that universal databases would be fairer to all citizens than the current discriminatory investigative databases, would aid exonerations of innocent people, would deter crime and would eliminate the invasion of privacy represented by mass-screens (or ‘dragnets’) and familial searching [73]. The rise of investigative genetic genealogy and the use (and abuse) of publicly accessible genetic data by law enforcement has been used to further strengthen arguments in favour of universal forensic databases [74]. Problems with BGA testing and the prediction of EVCs could be marshalled as yet an additional justification. Given Lewontin's own social activism and his commitment to building a better world [75], as well as his general scepticism about forensic genetics [14,76], it seems most unlikely that he would have signed up to universal databases, and there are certainly powerful arguments to be made against them [25]: they would be expensive, place disproportionate restrictions upon individual rights to privacy, treat the population as suspects (rather than citizens presumed innocent) and raise serious problems in navigating consent and its inevitable refusal by some. Since forensic databases operate at the level of nations, there would be thorny issues around the DNA profiling of visiting workers and tourists, and no doubt different nations would behave differently in this respect [25]. There is no reason to believe that the creation of universal databases would make criminal justice systems fairer for ethnic minorities [77].

How can the current situation be mitigated? There is a clear need for good practice in considering human classifications in imperfect but important forensic probability estimates. Labels matter, and should be used more carefully; this should include a nuanced consideration of admixture, rather than the shoe-horning of DNA donors into individual groups. It is promising that the field has woken up recently to the issue of ethics, and in particular to the question of the informed consent of participants in forensic population studies [78]. This suggests that the broader questions around how forensic genetics interacts with racial classifications and a public view of human difference should also be the subject of consideration and regular re-evaluation, rather than relying on tablets of stone from a previous era [19] representing empirical and arbitrary standards.

Finally, as with those who study and write about population genetics and genomics, there is a responsibility for the scientist who uses and reports on forensic prediction of ancestry and phenotypes to think carefully about the language, the narrative, and the message that they convey to the public [79].

## Data Availability

This article has no additional data.
